# Suppressor tRNAs at the interface of genetic code expansion and medicine

**DOI:** 10.3389/fgene.2024.1420331

**Published:** 2024-05-10

**Authors:** Aya Awawdeh, Alexander A. Radecki, Oscar Vargas-Rodriguez

**Affiliations:** Department of Molecular Biology and Biophysics, University of Connecticut School of Medicine, Farmington, CT, United States

**Keywords:** tRNA, translation, genetic code expansion, synthetic biology, premature termination codons, nonsense mutations, RNA therapeutics

## Abstract

Suppressor transfer RNAs (sup-tRNAs) are receiving renewed attention for their promising therapeutic properties in treating genetic diseases caused by nonsense mutations. Traditionally, sup-tRNAs have been created by replacing the anticodon sequence of native tRNAs with a suppressor sequence. However, due to their complex interactome, considering other structural and functional tRNA features for design and engineering can yield more effective sup-tRNA therapies. For over 2 decades, the field of genetic code expansion (GCE) has created a wealth of knowledge, resources, and tools to engineer sup-tRNAs. In this Mini Review, we aim to shed light on how existing knowledge and strategies to develop sup-tRNAs for GCE can be adopted to accelerate the discovery of efficient and specific sup-tRNAs for medical treatment options. We highlight methods and milestones and discuss how these approaches may enlighten the research and development of tRNA medicines.

## 1 Introduction

An estimated 10% of human genetic diseases are caused by nonsense mutations (or premature termination codons, PTCs) (Mort et al., 2008). PTCs introduce a stop codon within a gene’s protein-coding region, prematurely terminating gene translation and producing truncated, non-functional proteins. Several debilitating and life-threatening conditions, such as cystic fibrosis and Duchenne muscular dystrophy, are caused by nonsense mutations (Stenson et al., 2020). In addition to inherited mutations, PTCs can originate from somatic mutations, causing diseases like cancer (Zhang et al., 2021). Very few treatment options are available for patients suffering from PTC-related conditions. In recent years, suppressor tRNAs (sup-tRNAs) have regained notoriety as a promising therapeutic approach based on their ability to translate PTCs and restore protein synthesis (Porter et al., 2021; Dolgin, 2022; Lin and Glatt, 2022; Anastassiadis and Köhrer, 2023; Coller and Ignatova, 2024). The reinvigorated interest in sup-tRNAs is supported by exciting new evidence demonstrating their efficacy and safety in mouse models together with available RNA delivery strategies (e.g., lipid nanoparticles and adeno-associated virus) (Lueck et al., 2019; Wang et al., 2022; Albers et al., 2023). Despite recent progress, several challenges and knowledge gaps remain. Among them is the ability to design and engineer efficient and specific sup-tRNAs. Most sup-tRNAs tested for disease-related applications have been created by introducing suppressor anticodon sequences into native tRNAs (Temple et al., 1982; Panchal et al., 1999; Sako et al., 2006; Bordeira-Carriço et al., 2014; Lueck et al., 2019; Wang et al., 2022). While this approach yields tRNAs capable of suppressing PTCs, they tend to display poor translation efficiencies and specificity. Thus, developing new and more effective sup-tRNAs remains a fundamental area of research.

Coincidentally, for the past 20 years, research in genetic code expansion (GCE) has been at the forefront of sup-tRNA engineering. GCE is a powerful biotechnology that enables the synthesis of proteins with noncanonical amino acids at desired positions. GCE effectively increases the chemical diversity and functions of proteins (Chin, 2017). Notably, GCE applications have been successfully implemented in human-cultured cells and different model organisms, including whole animal models (Brown et al., 2018). The targeted incorporation of noncanonical amino acids via GCE requires an intentionally positioned PTC in the mRNA coding sequence of the protein of interest. Thus, sup-tRNAs are a central component of GCE as they mediate the noncanonical amino acid incorporation (Reynolds et al., 2017). The central role of sup-tRNAs in GCE has promoted significant work towards creating and enhancing sup-tRNAs, which has enriched our knowledge of sup-tRNA engineering (Kim et al., 2024). Notably, the advances in GCE have led to the development of robust and high-throughput platforms for the design, engineering, and artificial evolution of sup-tRNAs (Kim et al., 2024). Paradoxically, despite their shared goals of developing sup-tRNAs, a gap exists between the fields of GCE and tRNA therapeutics. Here, we aim to bring attention to the available information and tools that have emerged from GCE studies that can contribute to the discovery and advancement of tRNA-based medicines.

## 2 The universal role of tRNAs

**FIGURE 1:**
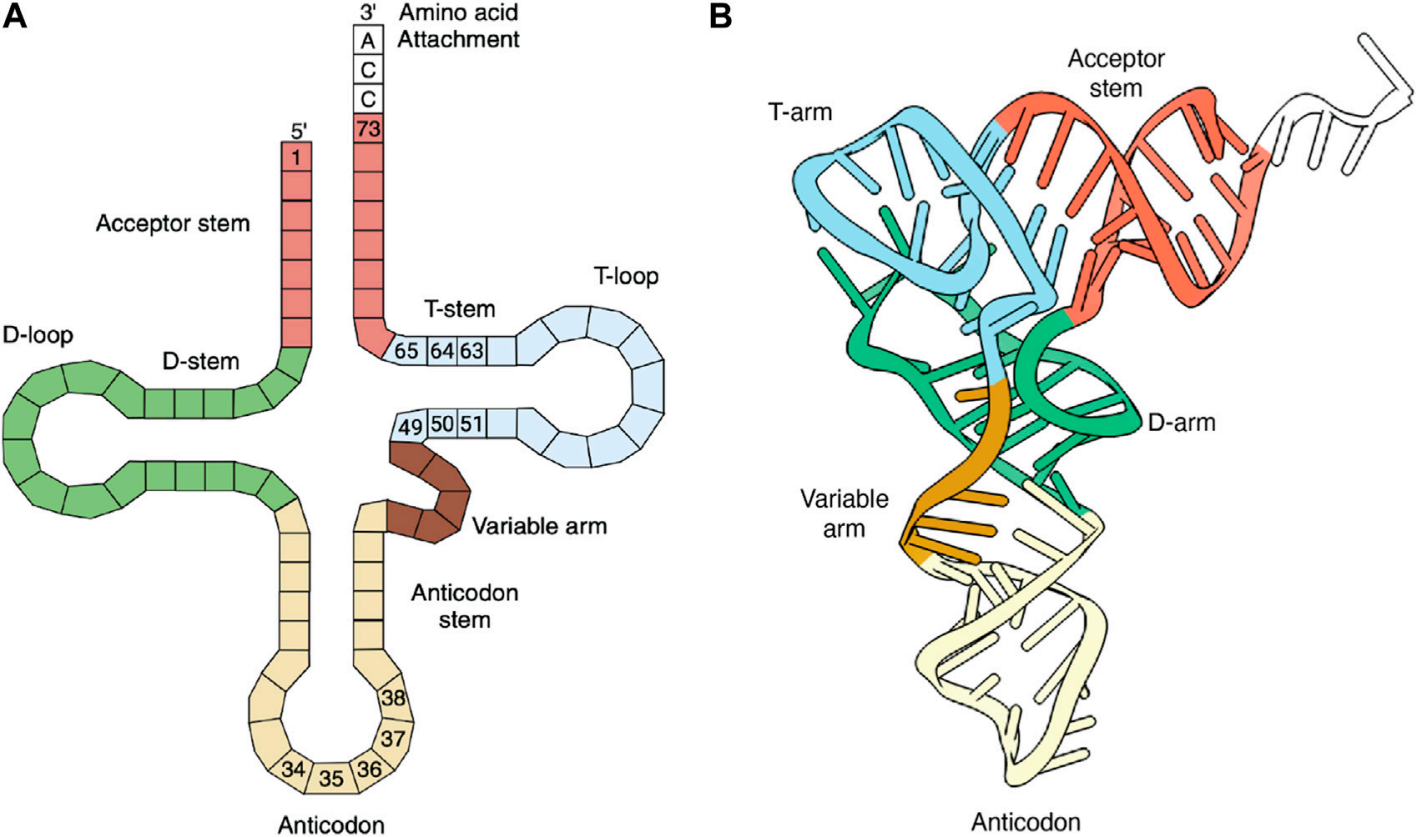
tRNA structure. **(A)** Secondary (cloverleaf) structure. The anticodon and elongation factor’s recognition bases are numbered. Bases 37 and 38 are known to increase PTC translation. **(B)** Tertiary (L-shaped) structure. **(A,B)** were created with BioRender.com and the tRNA crystal structure (PDB:1EVV), respectively.

**FIGURE 2:**
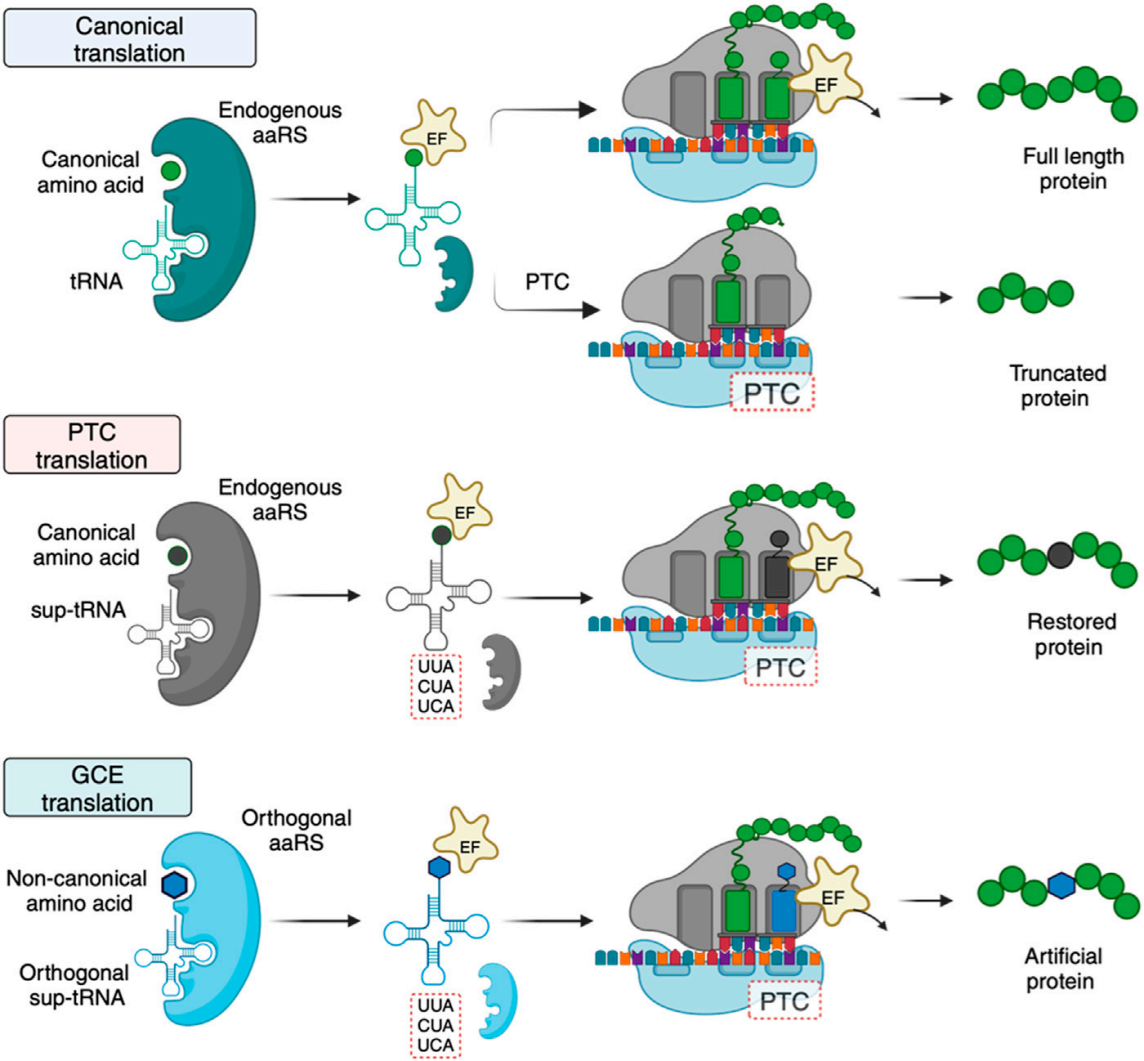
Mechanistic differences and requirements between canonical, PTC, and GCE translation. For GCE translation, an orthogonal tRNA-aaRS pair is required. The orthogonal pair does not interact with endogenous tRNAs and aaRSs. The orthogonality requirement is achieved by introducing a tRNA-aaRS pair from an organism distinct from the host species. Created with BioRender.com.

The principal role of tRNAs is to provide the ribosome with the amino acid building blocks for protein synthesis. tRNAs typically comprise 70–100 bases that fold into a strictly conserved L-shaped tertiary structure (Figure 1). With few exceptions, tRNAs are composed of 4–5 stems and 3–4 loops that form a cloverleaf secondary structure. During translation, tRNAs are ligated with their cognate amino acids by aminoacyl-tRNA synthetases (aaRSs) (Ibba and Söll, 2000). The elongation factor transports the resulting aminoacylated tRNAs to the ribosome, where codon-anticodon base pairing is established, and the incoming amino acid is incorporated into the nascent protein (Figure 2). Each of these steps involves highly choreographed interactions that contribute to the accurate translation of genetic information into proteins. Consequently, engineering effective sup-tRNAs requires individually and collectively considering the tRNA sequence or structural elements that define these interactions.

## 3 Distinct engineering considerations for sup-tRNAs

Sup-tRNAs for GCE and therapeutic applications are engineered to translate a targeted PTC with a desired amino acid. However, the requirements of the engineered sup-tRNAs differ. For GCE, the sup-tRNAs are introduced into an organism with a dedicated exogenous aaRS partner that only aminoacylates the sup-tRNA (Figure 2) (Vargas-Rodriguez et al., 2018). Importantly, the host cell’s aaRSs must not recognize the sup-tRNA. To meet this requirement, sup-tRNA-aaRS pairs in GCE are typically transplanted from organisms that are phylogenetically distant from the recipient species (Icking et al., 2024). For example, sup-tRNAs and aaRSs used in eukaryotic cells (including humans) usually have bacterial or archaeal origins (Italia et al., 2017b). In contrast, sup-tRNAs for therapeutic purposes are designed to be recognized by an endogenous aaRS, eliminating the requirement for a dedicated aaRS partner (Figure 2). This stark difference simplifies the development of therapeutic sup-tRNAs relative to GCE.

## 4 The intricacies of tRNA engineering: insights from GCE research

Efforts in GCE to optimize translation of PTCs with noncanonical amino acids have relied on foundational knowledge of the dynamic interaction of tRNAs with different translation factors, including the ribosome, aaRSs, and elongation factor. As a result, GCE studies have validated some of the early knowledge while uncovering and defining intricacies that can be exploited to improve sup-tRNA aminoacylation, delivery, and decoding. In this section, we describe the key basis of tRNA interactions with translation factors and how their manipulation has led to the design and engineering of improved sup-tRNAs in GCE.

### 4.1 Interactions with aaRSs

Like native tRNAs, ideal sup-tRNAs must be ligated with an amino acid by a specific aaRS. Most aaRSs select their cognate tRNA substrates via interactions with two of the tRNA’s structural features: the anticodon loop and the acceptor stem (Figure 1). A dedicated anticodon binding domain generally mediates the anticodon recognition, while the aminoacylation domain recognizes the acceptor stem (Giegé and Eriani, 2023). The identity of tRNAs for a particular amino acid is defined by these unique sets of tRNA-aaRS interactions, preventing cross-reactions between non-cognate tRNAs and aaRSs. Due to the stringent anticodon recognition, converting the anticodon sequence of a canonical tRNA into a suppressor sequence significantly decreases the interaction and affinity with the cognate aaRS. However, some aaRSs do not directly interact with tRNA anticodon or depend little on the anticodon bases (Giegé and Eriani, 2023). For example, leucyl- and seryl-tRNA synthetase do not recognize the anticodon, while arginyl- and tyrosyl-synthetase tolerate changes in their tRNA substrate’s anticodons. Consequently, sup-tRNAs (natural and engineered) generally originate from tRNAs whose cognate aaRS partners accept mutation in the anticodon.

Nonetheless, mutations in the anticodon can also affect aaRS’s catalytic function via distal communication within the tRNA manifested in its 3′-end (Ibba et al., 1996; Uter and Perona, 2004). The weaker tRNA-aaRS interaction results in low aminoacylation efficiency, a common feature of most engineered tRNAs (Vargas-Rodriguez et al., 2018). The intricacies of the tRNA-aaRS interactions go beyond the direct role of tRNA bases in binding and catalysis. Even changes in tRNA regions that do not directly contact the aaRS may influence aminoacylation (Giegé and Eriani, 2014). Finally, changes in the anticodon can cause unintended cross-reactions between aaRSs (Normanly et al., 1990; Zheng et al., 2017; Giegé and Eriani, 2023; Osgood et al., 2023). In bacteria, a tRNA^Trp^ with CUA, but not with UCA, is aminoacylated by glutaminyl-tRNA synthetase (Italia et al., 2017a). This cross-reactivity with noncognate aaRSs is also observed in engineered human sup-tRNAs (Wang et al., 2022). The complex interplay between tRNAs and aaRSs complicates the rational design of sup-tRNAs.

Efforts in GCE have been aimed at overcoming these limitations. Using high-throughput screening and selection engineering platforms, sup-tRNAs with improved interactions with their aaRS partners that increase tRNA aminoacylation levels have been obtained (Wang and Schultz, 2001; Anderson et al., 2004; Guo et al., 2009; Chatterjee et al., 2012; Chatterjee et al., 2013b; Javahishvili et al., 2014; Jewel et al., 2023). Notably, the enhanced sup-tRNA-aaRS interactions are achieved by mutations in the acceptor stem, suggesting that fine-tuning interactions in other tRNA regions can compensate for a decrease in the anticodon binding. Similarly, sup-tRNAs have also been optimized to prevent aminoacylation by noncognate aaRSs (Zhang et al., 2017; Grasso et al., 2022). This knowledge can aid and guide the sup-tRNA engineering efforts for therapeutic applications.

### 4.2 Interactions with elongation factor

In contrast to aaRSs, the elongation factor (EF-Tu in bacteria and eEF1A in eukaryotes) must interact with all aminoacylated tRNAs (except for initiator tRNA^Met^, which is recruited to the ribosome by initiation factors). This process involves the uniform recognition of tRNAs with diverse structural and sequence features attached to amino acids with different side chains. EF-Tu achieves uniform binding affinities using a compensatory thermodynamic mechanism in which the amino acid moiety and the tRNA body additively contribute to the overall binding (LaRiviere et al., 2001; Uhlenbeck and Schrader, 2018). As a result, EF-Tu exhibits a wide range of binding affinities toward tRNA isoacceptors. The tRNA affinity is determined by three base pairs located in the T-stem (49:65, 50:64, and 51:63) (Figure 1) (Schrader et al., 2009). tRNAs with G51:C63, C50:G64, and G49:U65 T-stems are preferred EF binding partners (Schrader et al., 2009, 2011). Notably, GCE studies have demonstrated that optimizing the T-stem sequence of the translation efficiency sup-tRNAs can drastically improve suppression efficiency (Guo et al., 2009; Young et al., 2010; Fan et al., 2015; Thyer et al., 2015; Serfling et al., 2018). For example, mutations in the T-stem of pyrrolysine tRNA (a natural sup-tRNA) increase suppression efficiency by 5-fold (Fan et al., 2015). However, defining the optimized T-stem for a particular sup-tRNA may require exploring permutations that consolidate the optimal binding to the elongation factor. Some tRNAs may have an inherently optimal T-stem, preventing further improvement (Jewel et al., 2023). This knowledge was recently applied to the design of human sup-tRNAs, confirming the critical role of the elongation factor in suppression efficiency (Albers et al., 2023). This study also corroborated that EF-Tu and EF1A share the tRNA T-stem recognition (Albers et al., 2023).

### 4.3 Interactions with the ribosome

The elongation factor delivers tRNAs through complex interactions with the ribosome that facilitate the tRNA anticodon to base pair with the mRNA. The formation of correct Watson-Crick interactions triggers a local conformational rearrangement mediated by ribosomal RNA bases A1492, A1493, and G530, promoting the selection of cognate tRNAs (Rodnina, 2023). Recent structural studies of the ribosome reveal that this interaction network and the tRNA selection mechanism in the A-site are maintained in natural and artificial sup-tRNAs (Fischer et al., 2016; Albers et al., 2021; Hilal et al., 2022; Prabhakar et al., 2022). However, the movement of sup-tRNAs through the ribosome is moderately slower than native tRNAs (Prabhakar et al., 2022). In addition to the ribosomal structural features in the A-site, conserved base pairs in the tRNA anticodon stem may also contribute to establishing a faithful codon-anticodon base pairing (Ledoux et al., 2009; Murakami et al., 2009; Shepotinovskaya and Uhlenbeck, 2013). Enhancing these interactions has been proven to improve translation of targeted codons. For example, a mutation that optimizes the anticodon stem of a sup-tRNA increases suppression efficiency by 2-fold (Anderson and Schultz, 2003; Chatterjee et al., 2013a). Fine-tuning the anticodon stem-loop can improve affinity and translation efficiency (Katoh and Suga, 2024). Other anticodon structural and sequence features can also facilitate PTC suppression (Rogerson et al., 2015). For example, adenosines at positions 37 and 38 substantially increase suppression efficiency (Kleina et al., 1990; Normanly et al., 1990; Wu et al., 2004; Englert et al., 2017).

tRNA modifications also play a crucial role in mRNA decoding. In GCE applications, anticodon modifications were shown to influence suppression efficiency of two distinct sup-tRNAs in bacteria (Baldridge et al., 2018; Crnković et al., 2018). Although the molecular mechanism remains unknown, these studies underscore the importance of considering the fundamental role of modifications in sup-tRNA engineering.

Another notable observation in GCE is that some tRNA scaffolds are more suitable for a particular stop codon. For example, the natural suppressor tRNA^Pyl^ is more efficient at translating its cognate codon UAG than UGA. It can also mistranslate UAG, albeit with two times lower efficiency (Morosky et al., 2023). Moreover, intrinsic tRNA elements contribute to codon specificity, as shown for a synthetic sup-tRNA for selenocysteine with CUA anticodon that mistranslates UGA codons with similar efficiency as the cognate UAG (Morosky et al., 2023). However, the same sup-tRNA with UCA anticodon is specific for UGA. Understanding the molecular basis for these codon-specificity behaviors requires further investigation. Nonetheless, these observations underscore the intricacies of sup-tRNAs during decoding.

## 5 Translation deficiencies of sup-tRNA therapeutic candidates

Like in GCE applications, most engineered human sup-tRNAs used to translate disease-causing PTCs fail to fully restore the synthesis of the target proteins to wild-type levels, achieving, on average, less than 40% suppression in recent studies (Bordeira-Carriço et al., 2014; Lueck et al., 2019; Wang et al., 2022; Albers et al., 2023; Blomquist et al., 2023; Bharti et al., 2024). Notably, varying suppression efficiencies are observed for sup-tRNAs with different identities and nonsense anticodons. This may be due to the suboptimal interactions with their interacting partners, which likely hinder sup-tRNAs’ decoding capacity. As discussed in the previous section, poor aminoacylation by aaRSs, sup-tRNA delivery to the ribosome by EF1A, and interactions with the mRNA and ribosome during decoding contribute to the overall efficiency of synthetic human sup-tRNAs. However, the specific contribution of each of these steps is generally obscured in most studies because the total output of a reporter protein is usually used to determine translation efficiency. Thus, unraveling the molecular mechanisms determining PTC translation by sup-tRNA will require additional detailed biochemical studies. These mechanistic details of PTC decoding will better inform engineering efforts to enhance sup-tRNAs. For example, tuning the interaction with EF1A improves sup-tRNA activity (Albers et al., 2023).

## 6 Existing platforms for sup-tRNA design and development

Developing and improving sup-tRNAs have been a major focus of GCE, resulting in several pioneering approaches for sup-tRNA engineering (Wang et al., 2001; Wang and Schultz, 2001; Guo et al., 2009; Maranhao and Ellington, 2017; DeBenedictis et al., 2021). Although efforts have focused on enhancing the interaction between tRNAs and their cognate aaRSs to increase aminoacylation levels, these platforms can be adapted to screen and select sup-tRNA variants with improved EF affinity or decoding efficiency (Wang and Schultz, 2001; Guo et al., 2009; Rogerson et al., 2015; DeBenedictis et al., 2021). These platforms integrate combinatorial approaches to create large tRNA mutant libraries that can be selected or screened in a high-throughput fashion with sensitive reporter proteins (Kim et al., 2024). A potential limitation is that these systems are mostly based on *Escherichia coli*. Therefore, retrofitting them to enable human sup-tRNA engineering will be needed. Nonetheless, given their robustness and tRNA sequence space that can be explored, efforts to repurpose these platforms may provide invaluable insights. Human aaRSs that function in *E*. *coli* cells and do not cross-react with bacterial tRNAs are ideal candidates to pursue this goal.

While bacterial platforms remain the primary avenue for tRNA engineering, recent work established the virus-assisted directed evolution of tRNA (VADER) (Jewel et al., 2023; Jewel et al., 2024). VADER facilitates the screening of >60,000 unique tRNA variants in human cultured cells, offering a novel avenue for rapid human sup-tRNA discovery. Adapting VADER for human sup-tRNA engineering will require establishing sensitive reporters that signal the incorporation of the intended amino acid. This will avoid the need for mass spectrometry analyses that hinder the speed of sup-tRNA discovery.

A limitation of most tRNA engineering platforms is that they depend on producing a protein reporter, reflecting the combined outcome of aminoacylation and decoding. Thus, investigating how changes in tRNA affect aminoacylation and decoding separately will allow us to discern their direct contribution to PTC translation. This knowledge can aid in designing better engineering strategies.

## 7 Discussion

Sup-tRNAs represent a transformative pharmacological opportunity to treat human genetic diseases. The prospect of therapeutic sup-tRNAs requires a better understanding of the mechanism of PTC translation, how synthetic tRNAs are metabolized, and delivery strategies (Coller and Ignatova, 2024). In the context of PTC translation, developing potent sup-tRNAs is critical and remains a fundamental area of research. The translation proficiency of sup-tRNAs can determine dosage indications. Moreover, effectively rescuing diverse pathogenic PTCs will depend on sup-tRNAs’ ability to translate PTCs in different positions within the target mRNA. Sup-tRNAs are known to display varying decoding efficiencies based on the location of PTC within the mRNA coding region (Bossi, 1983; Atkinson and Martin, 1994; Bharti et al., 2024). Another consideration for sup-tRNA therapies is the identity of the amino acid they carry. To faithfully restore protein synthesis from a PTC-containing gene, the sup-tRNA must be aminoacylated with the corresponding amino acid. The PTC-related diseases involve all three stop codons, which generally emerge from mutations of Arg, Gln, Ser, Glu, Tyr, Lys, Trp, Gly, Leu, and Cys codons (Stenson et al., 2020). Correction of each PTC would require a panel of sup-tRNAs with identities for each amino acid. Only a few families of sup-tRNAs with Tyr, Ser, Trp, Gly, and Arg identities with varying translation efficiencies for specific PTCs have been validated in animal models (Lueck et al., 2019; Wang et al., 2022; Albers et al., 2023). These tRNAs have been generated using native human tRNAs as scaffolds. However, this strategy may not be suitable for developing potent sup-tRNAs or require further engineering.

The innovation in GCE research can help guide the optimization of existing therapeutic sup-tRNA candidates and the creation of new ones. The implementation of GCE applications has gained substantial knowledge of how to design and engineer tRNAs. As discussed earlier, GCE has provided important mechanistic insights into the key elements that should be considered to improve sup-tRNA aminoacylation and decoding. GCE has also validated approaches to enhance sup-tRNA expression in mammalian cells (Zheng et al., 2017; Brown et al., 2018). The information gained regarding promoter designs and tRNA gene arrangement can be essential for establishing efficacious tRNA delivery using an adeno-associated virus or lipid nanoparticles (Zuko et al., 2021; Wang et al., 2022; Albers et al., 2023). Finally, given the need to treat different pathogenic PTCs, GCE has demonstrated that using tRNAs from other biological sources (i.e., species) in human cells can be a suitable and safe option for obtaining a panel of diverse sup-tRNAs capable of carrying desired amino acids. Integrating this knowledge collectively during human sup-tRNA engineering can accelerate the discovery of more proficient candidates.
